# Supplementary material for: HCV Activates Somatic L1 Retrotransposition—A Potential Hepatocarcinogenesis Pathway
Source: Cancers (Basel). 2021 Oct 11;13(20):5079. doi: 10.3390/cancers13205079 (PMC8533982; doi:10.3390/cancers13205079)
Supplement: Supplementary file 1 [file cancers-13-05079-s001.zip › cancers-1366777-supp-final.pdf]

Supplementary Materials

HCV Activates Somatic L1 Retrotransposition—A Potential Hepatocarcinogenesis Pathway

Praveen D. Sudhindar, Daniel Wainwright, Santu Saha, Rachel Howarth, Misti McCain, Yvonne Bury, Sweta S. Saha, Stuart McPherson, Helen Reeves, Arvind H. Patel, Geoffrey J. Faulkner, John Lunec and Ruchi Shukla

1. Materials and Methods

1.1. DNA Damage Induction by Gemcitabine Treatment To Assess ATR and Chk1 Inhibitors Efficacies

Huh7 cells were seeded in 12-well plates to attain approximately 90% confluency the next day. Cells were pre-treated or not with ATRi (VE-821, 1 μM) and CHK1i (SRA737, 1 μM) for 30 min, followed by treatment with Gemcitabine (100 nM) for 4 h. Finally, the cells were harvested for protein extraction using PhosphoSafe lysis buffer (Merck Millipore, Bedford, MA, USA, 71296).

1.2. DNA Damage Induction via Doxorubicin Treatment To Assess ATM Inhibitor Efficacy

HepG2 cells were maintained in RPMI-1640 (Sigma, St. Louis, MO, USA) media supplemented with 10% foetal bovine serum, 1% glutamine and 1% penicillin-streptomycin. Cells were seeded in 6-well plates to attain approximately 90% confluency the next day. Wells were pre-treated or not with ATMi (KU-55933, 10 μM) for 30 min, followed by treatment with 100 nM of doxorubicin and harvested 24 h after treatment for protein extraction using PhosphoSafe lysis buffer.

1.3. Western Blot Analysis

Western immunoblotting of whole cell lysates was performed as described previously [31]. The primary antibodies used were anti-pChk1-ser345 (1:1000, Polyclonal anti-rabbit, Abcam, Cambridge, UK, ab58567), anti-pChk1-ser296 (1:1000, Monoclonal anti-rabbit, Abcam ab79758), p-p53-ser 15 (1:1000, polyclonal anti-rabbit, Santa Cruz, Dallas, TX, USA, SC-101762), p53 (mouse monoclonal, DAKO, M7001) and anti-GAPDH (Rabbit monoclonal, Sigma, SAB2108266).

1.4. Genomic DNA Extraction, PCR and Agarose Gel Electrophoresis

Genomic DNA was extracted using the DNeasy Blood and Tissue Kit (GmbH, Hilden, Germany, 69504) as per the manufacturer's protocol and DNA concentration was measured via the nanodrop method. PCR was carried out to assess GFP integration using Platinum Green Hot start 2× PCR master mix (Invitrogen, Carlsbad, CA, USA, 13001013) with FWD: 5′-GAAGAACGGCATCAAGGTGAAC-3′ and REV: 5′-GGTGCTCAGGTAGTGGTTGTC-3′ primers. 18S was amplified to be used as a loading control with primers FWD: 5′-GTAACCCGTTGAACCCCATT-3′ and REV 5′-CCATCCAATCGGTAGTAGCG-3′. The PCR products were subjected to agarose gel electrophoresis and the bands were imaged by exposing them to UV light using Biorad ChemiDoc imaging system (Bio-Rad Laboratories, California, USA).

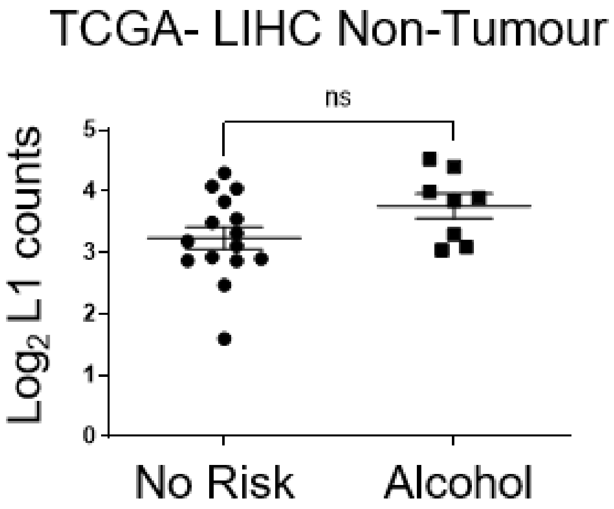

Figure S1. Graph representing the normalised L1 transcript count in the non-tumour liver of hepatocellular carcinoma (HCC) patients with indicated aetiologies from the cancer genome atlas liver hepatocellular carcinoma (TCGA-LIHC) study; no risk = no history of any known HCC risk factors. ns = non-significant ($p > 0.05$) by unpaired t-test.

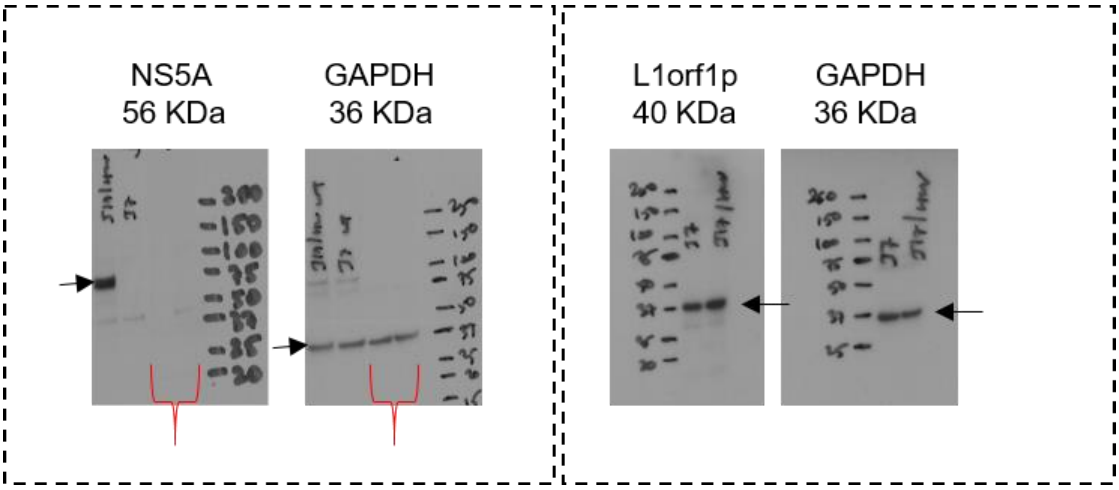

Figure S2. Whole X-ray film scans of Western blot analysis of whole cell lysates of Huh7 (J7) and Huh7-J17 (J17/HCV) cells with indicated antibodies. Membranes were reprobed for Glyceraldehyde 3-phosphate dehydrogenase (GAPDH) to determine protein loading. Expected molecular weights are indicated below each protein. Lanes marked as red are with lysates for another project. Key: NS5A = nonstructural protein 5A; L1orf1p = L1 open reading frame 1 encoded protein.

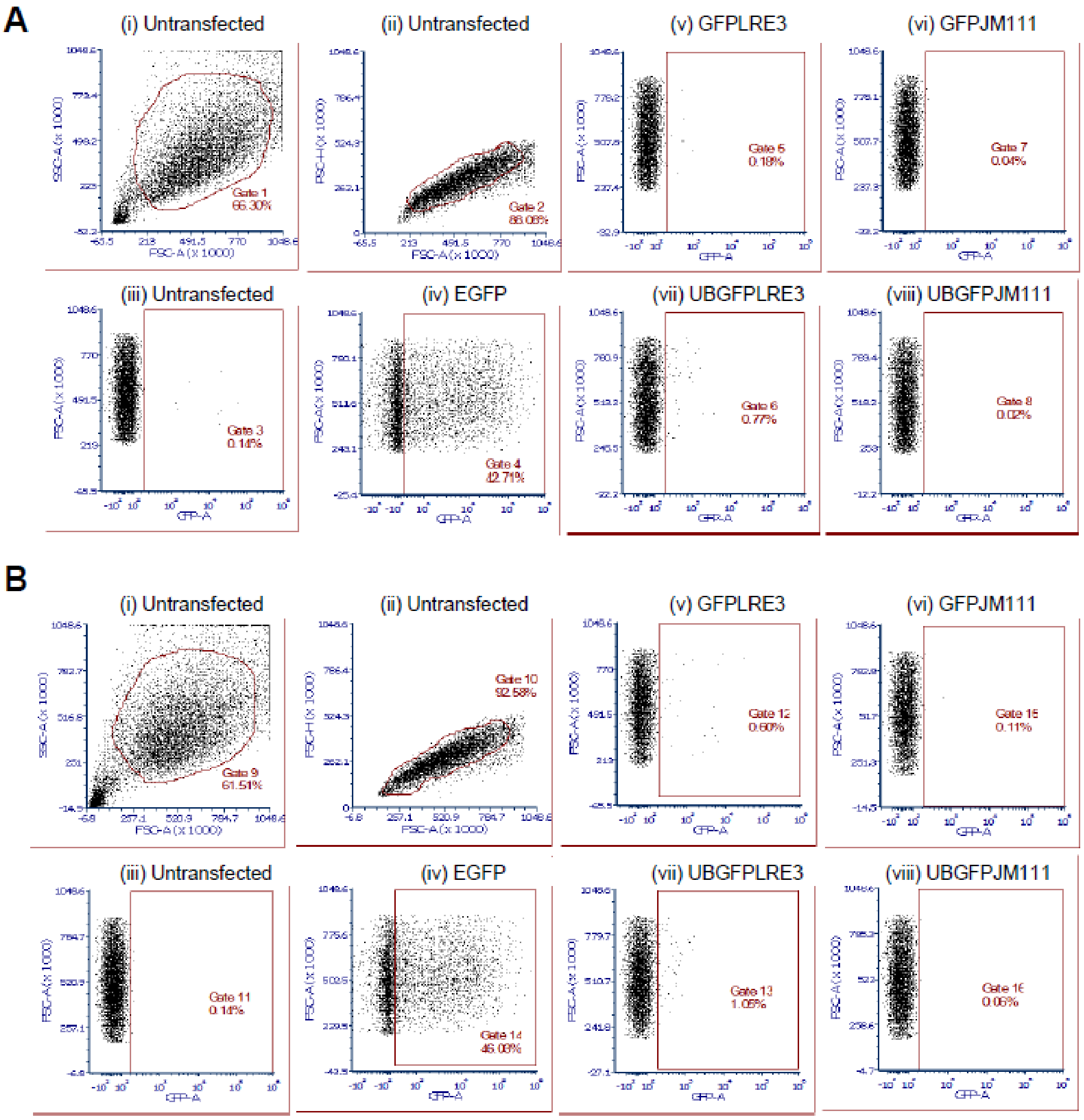

Figure S3. Enhanced Green Fluorescent Protein (EGFP)-based retrotransposition assay gating strategy for fluorescence activated cell sorting. EGFP-positive cells were selected by setting a gate on non-transfected cells to remove the background debris signal (FSC-A SSC-A plot **i**) and then selecting for the single cell population (FSC-A FSC-H plot **ii**). Then, a gate on the single cell population was set on non-transfected cells to set the auto fluorescence of the cells in the BL1 channel (plot **iii**). The shift in fluorescence was then assessed for EGFP plasmid transfected cells as a positive control (plot **iv**) and the level of retrotransposition was assessed in the GFPLRE3 and UBGFPLRE3 transfected cells (plots **v** and **vii**) and respective JM111 transfected cells (plots **vi** and **viii**) for (**A**) Huh7; and (**B**) Huh7-J17. Key: FSC = forward scatter; SSC = side scatter; A = area, H = Height.

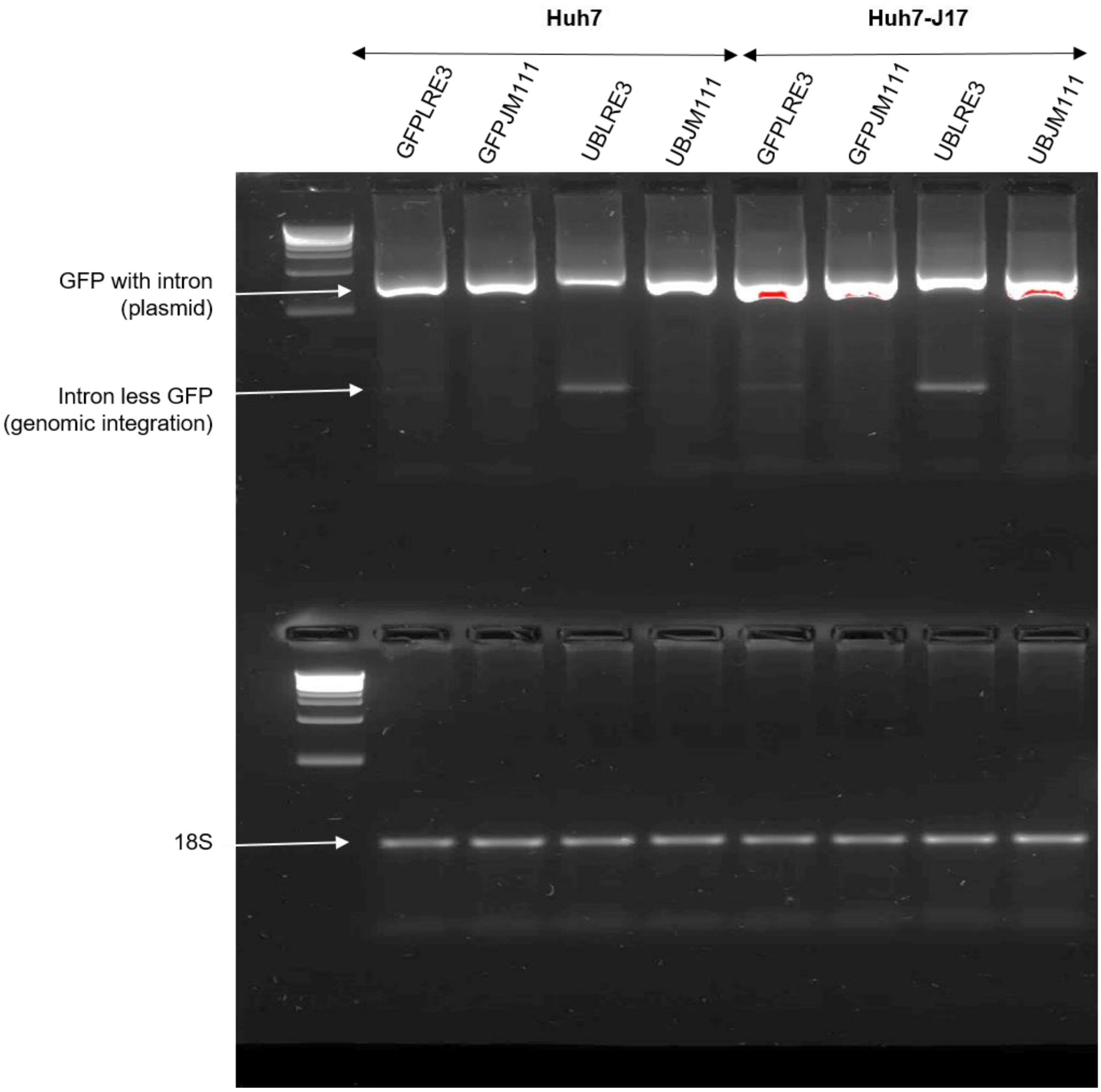

Figure S4. Enhanced Green Fluorescent Protein (EGFP)-based retrotransposition assessment via genomic polymerase chain reaction (PCR), followed by agarose gel electrophoresis**.** Indicated cell lines were transfected with GFP-LRE3 or UB-LRE3 (GFP-LRE3 under ubiquitin (UB) promoter) or corresponding mutant JM111 plasmids. Five days after transfection, genomic DNA was isolated and PCR was carried out with primers across the Green Fluorescent Protein (GFP) intron. The top band in the top gel indicates GFP amplification from the LRE3/JM111 plasmids (red signal within the bands denotes signal saturation), whereas the bottom band in the top gel indicates the intronless GFP and is visible only in cells transfected with LRE3 plasmids. The bottom gel represents the amplification of the 18S genomic region and is used as a loading control of the genomic DNA.

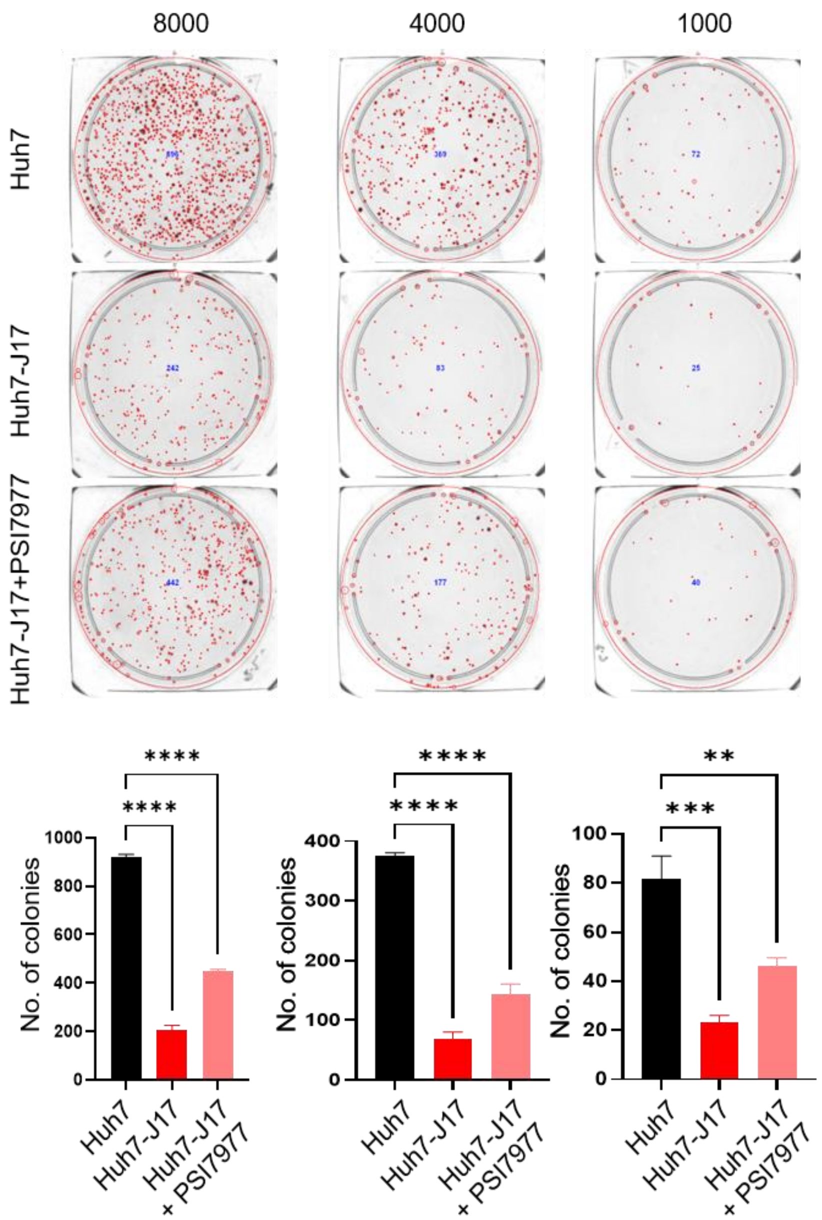

Figure S5. Plates representing colonies formed 2 weeks after the blasticidin selection of indicated cells transfected with pcDNA6.1-blast plasmid as visualised via crystal violet staining. Image is representative of 3 technical repeats. Four days after transfection, the transfected cells were harvested via trypsinisation and seeded in 10-cm dishes to start the selection. Numbers on top represent initial cells seeded. Numbers written within the plates represent colony count obtained by the colony counter. Graphs at the bottom represent the number of colonies in each cell line in respective seeding conditions. Non-transfected controls were run alongside each cell line and no colonies were obtained in any of the non-transfected controls. **$p < 0.01$, ***$p < 0.001$, ****$p < 0.0001$. One-way ANOVA with multiple comparisons using Huh7 as the control.

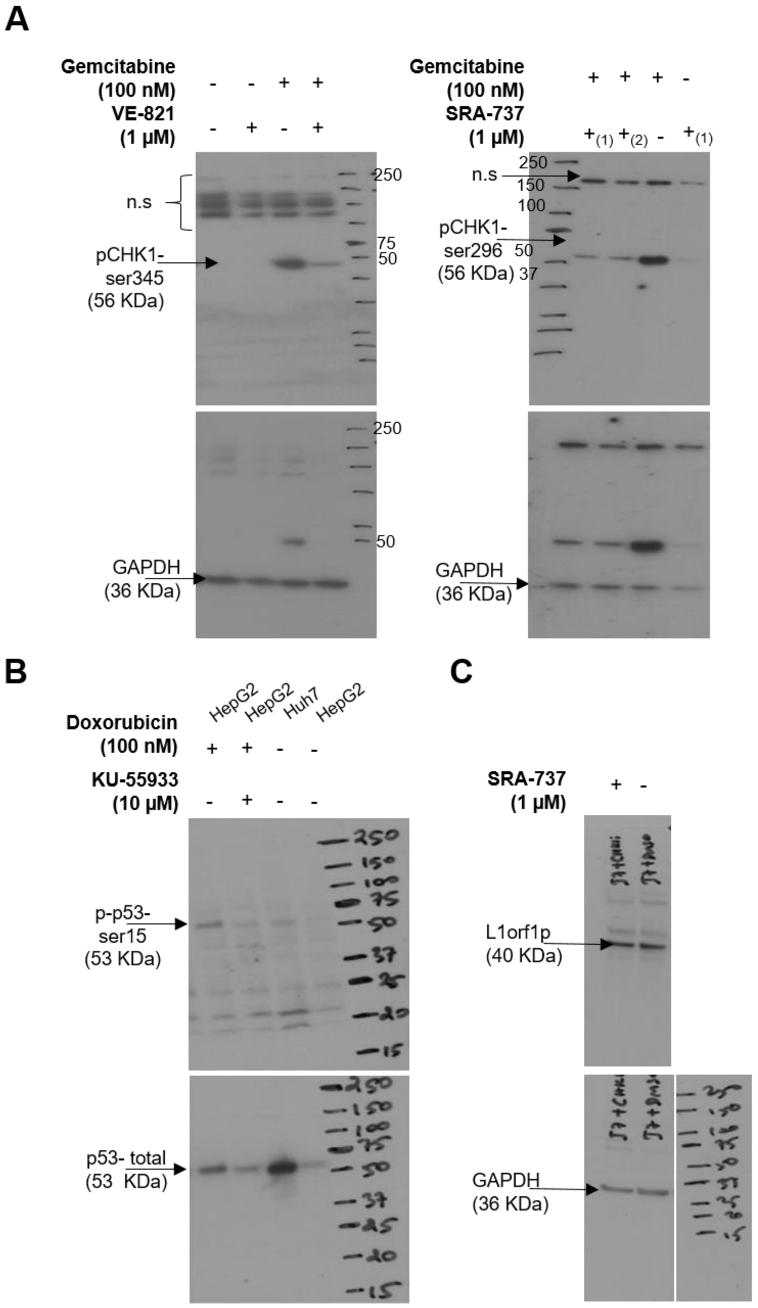

Figure S6. Assessment of the effectiveness of DNA Damage Response (DDR) inhibitors**.** DNA damage was induced by gemcitabine treatment (100 nM) in the presence or absence of indicated inhibitors in Huh7 cells and cell lysates were prepared 4 h after the induction to evaluate levels of phosphorylated-CHK1 as a surrogate for the ATR-Chk1 pathway activation. Bottom panel shows the membranes reprobed for GAPDH without stripping to assess protein loading. n.s. = nonspecific bands; 1 and 2 corresponds to biological replicate lysates (A). Since Huh7 cells are mutant for p53, DNA damage was induced via doxorubicin treatment (100 nM) in HepG2 cells (wild-type p53 cell line) in the presence or absence of ATMi (KU-55933) and cell lysates were prepared 24 h later to evaluate phosphor-p53 ser15. Bottom panel shows the membrane reprobed for total p53 after stripping. Untreated Huh7 cells' lysate was used as a positive control for total p53; accumulated p53 in Huh7 cells confirmed mutant p53 status (B). Huh7 cells were treated with 1 μM SRA-737 (Chk1i) or equivalent amount of DMSO as a vehicle control for 48 h and then whole cell lysates were prepared to evaluate L1orf1p expression. Bottom panel shows the membranes reprobed for GAPDH after stripping to assess protein loading (C). Key: ATMi = Ataxia Telangiectasia Mutated kinase inhibitor; ATRi = Ataxia-Telangiectasia and Rad3-related kinase inhibitor; CHK1i = Checkpoint kinase 1 inhibitor; p53 = tumour suppressor protein 53; GAPDH = Glyceraldehyde 3-phosphate dehydrogenase.

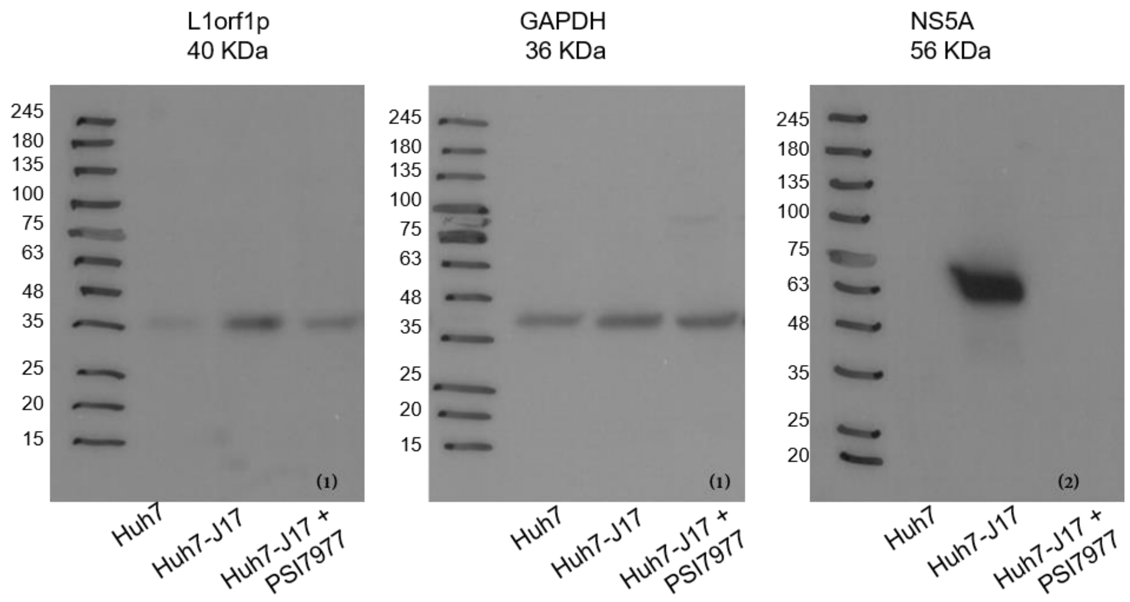

Figure S7. Whole X-ray film scans of Western blot analysis of whole cell lysates of Huh7, Huh7-J17 and Huh7-J17+PSI7977 cells with indicated antibodies. Gels for membrane 1 and 2 were run in parallel with same protein lysates. Membrane 1 was reprobed for GAPDH to determine protein loading. Expected molecular weights are indicated below each protein. Key: L1orf1p = L1 open reading frame encoded protein; NS5A = nonstructural protein 5A; GAPDH = Glyceraldehyde 3-phosphate dehydrogenase.

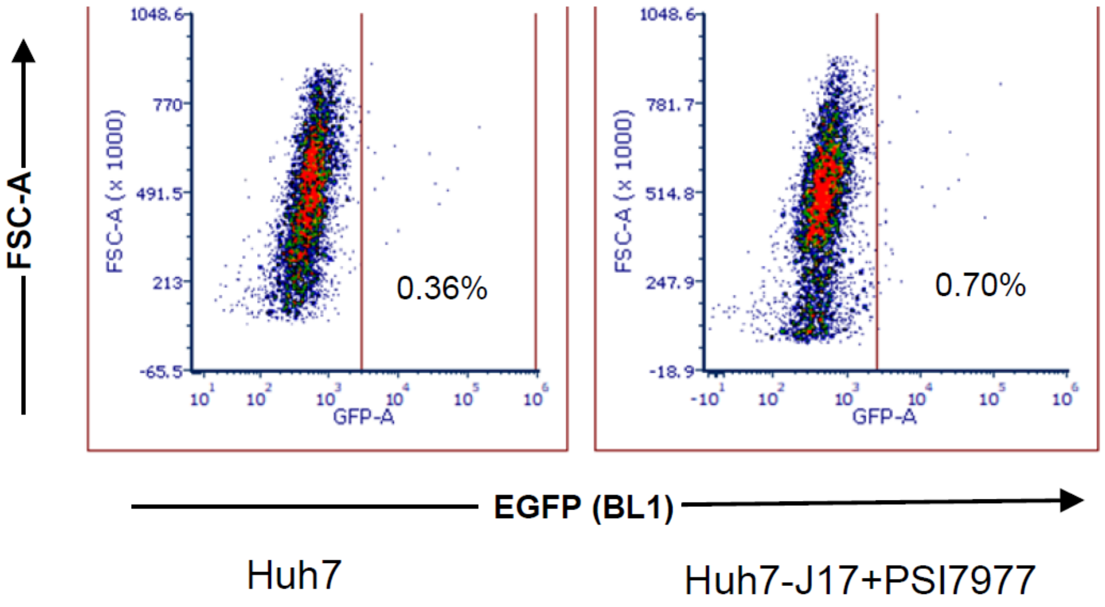

Figure S8. Enhanced Green Fluorescent Protein (EGFP)-based retrotransposition assay in indicated cells analysed by Fluorescence Activated Cell Sorting. Cells were transfected with UBGFPLRE3 plasmid and analysed 5 days after transfection to detect EGFP-positive cells, indicating successful retrotransposition. Dot blots represent cells plotted as Forward Scatter Area (FSC-A) versus BL1 channel for EGFP detection, vertical red line defines positive gating and number within the plot represents % positive cells.

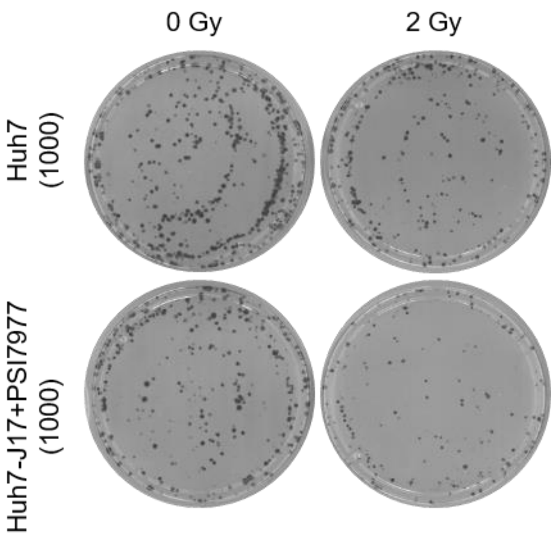

Figure S9. DNA damage repair response of indicated cell lines. Plates representing colonies formed without any treatment (0 Gy) and after cells were exposed to 2 Gy X-ray radiation as visualised by the crystal violet stain. Image is representative of 2 independent repeats performed in duplicates. Numbers represent initial seed counts.

© 2021 by the authors. Licensee MDPI, Basel, Switzerland. This article is an open access article distributed under the terms and conditions of the Creative Commons Attribution (CC BY) license (http://creativecommons.org/licenses/by/4.0/).
