# Supplementary figures and images for: HCV Activates Somatic L1 Retrotransposition—A Potential Hepatocarcinogenesis Pathway
Source: Cancers (Basel). 2021 Oct 11;13(20):5079. doi: 10.3390/cancers13205079 (PMC8533982; doi:10.3390/cancers13205079)

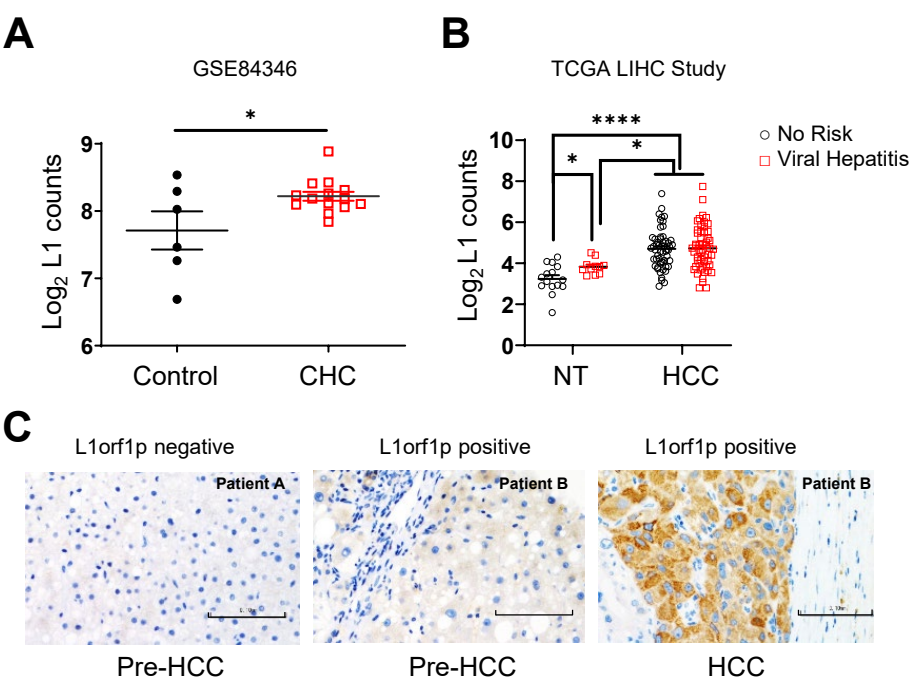

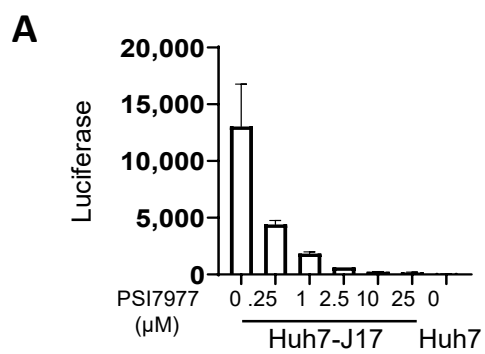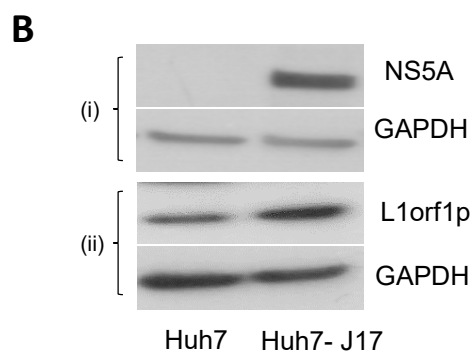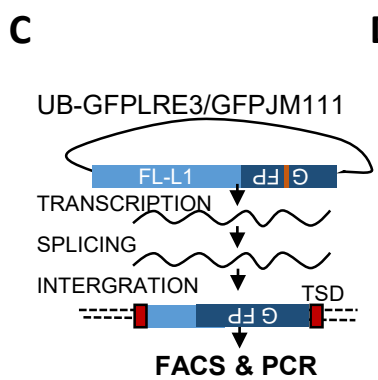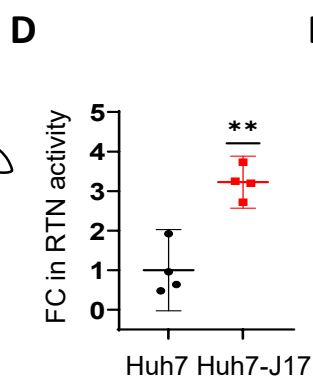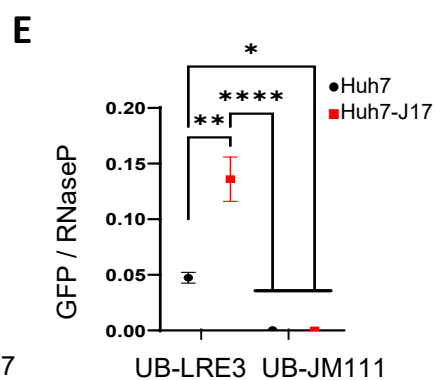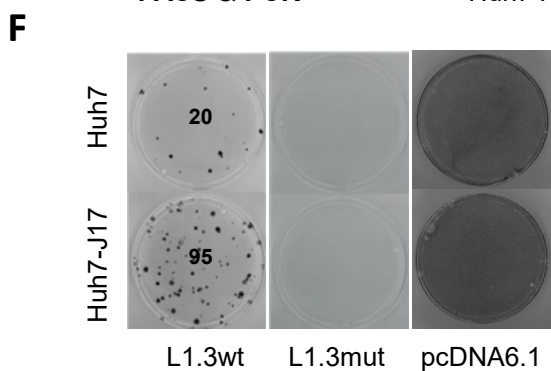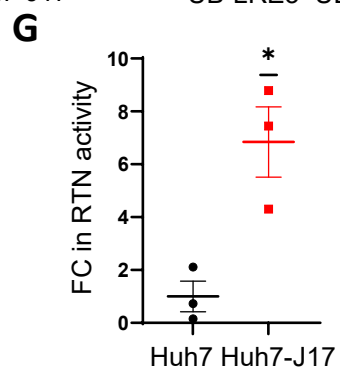

**A**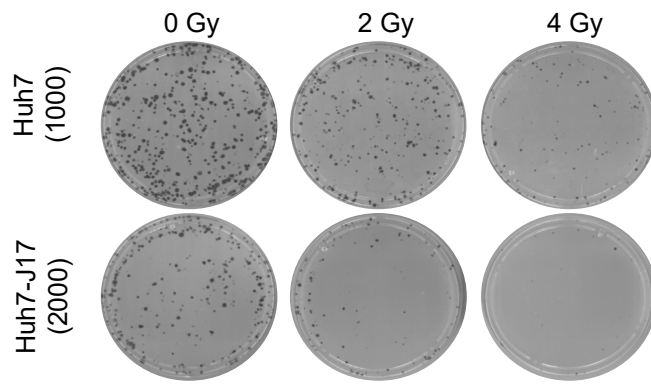**B**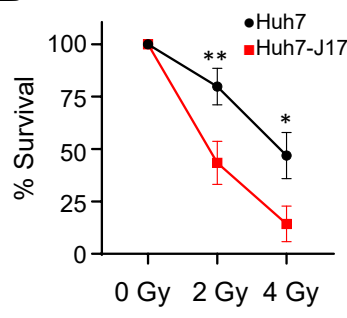**C**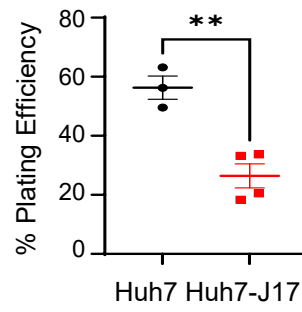

**A**

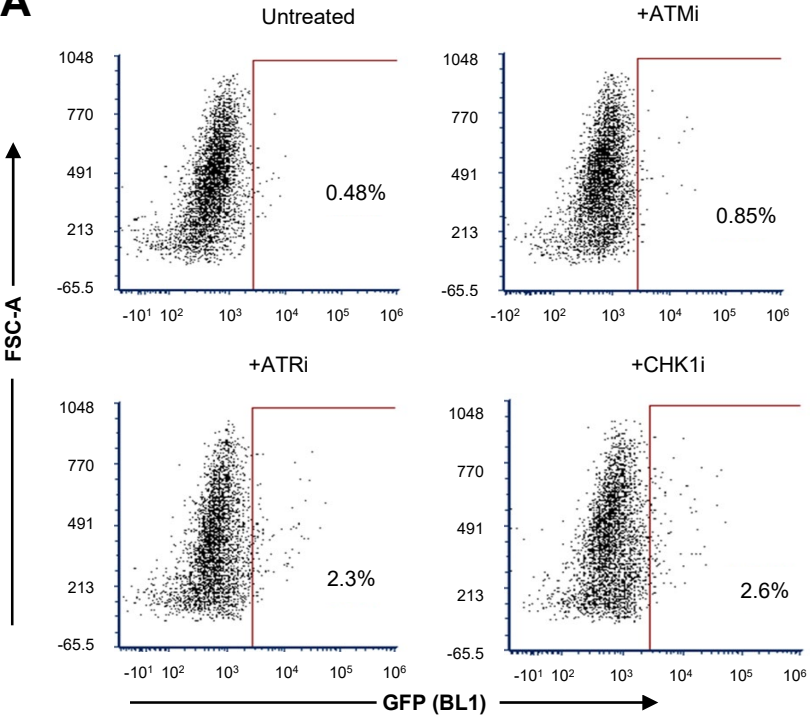

**B**

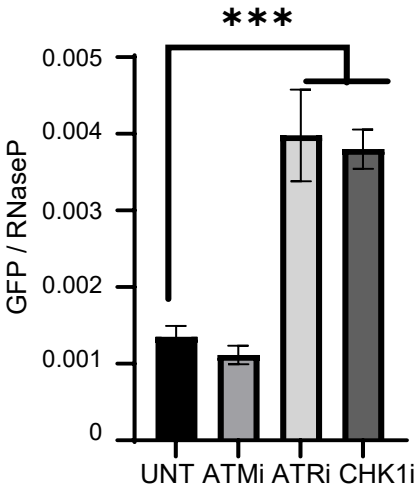

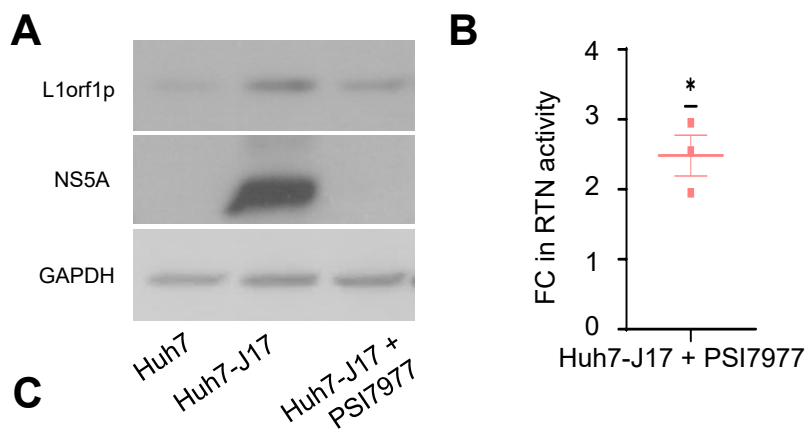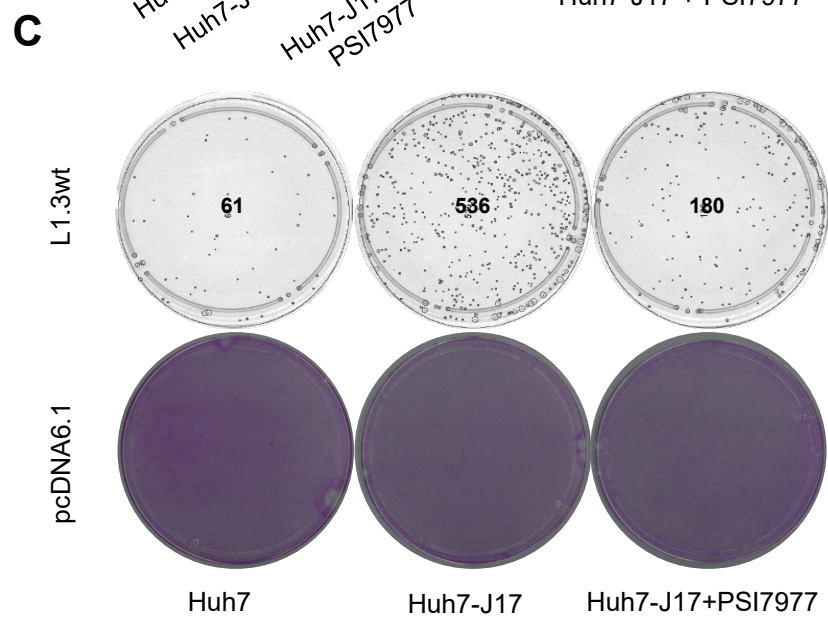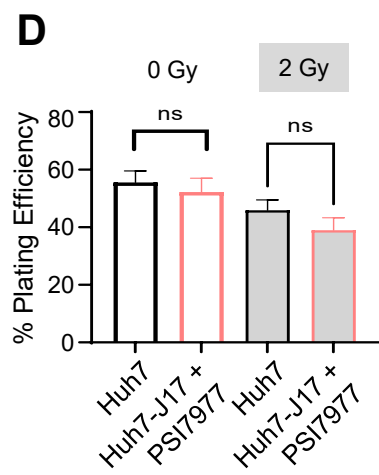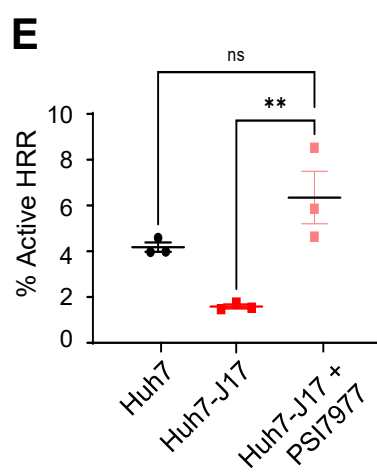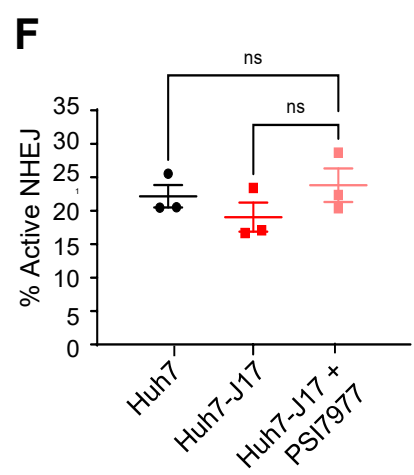

TCGA- LIHC Non-Tumour

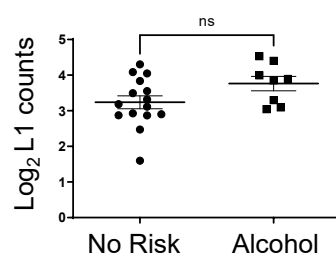

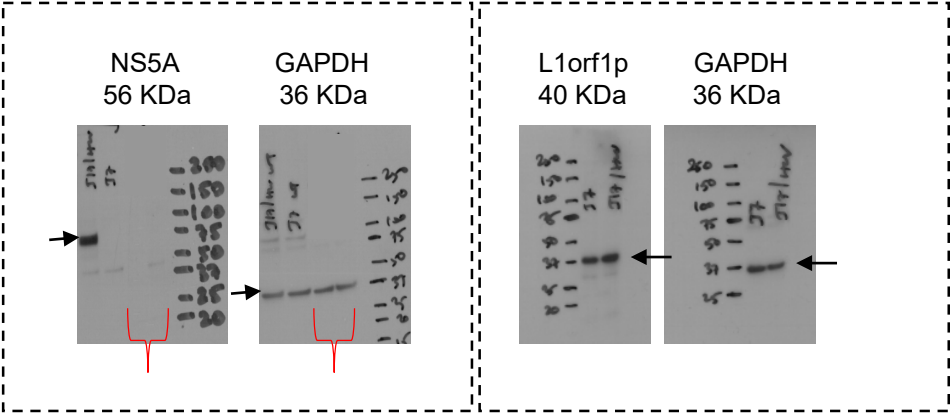

A

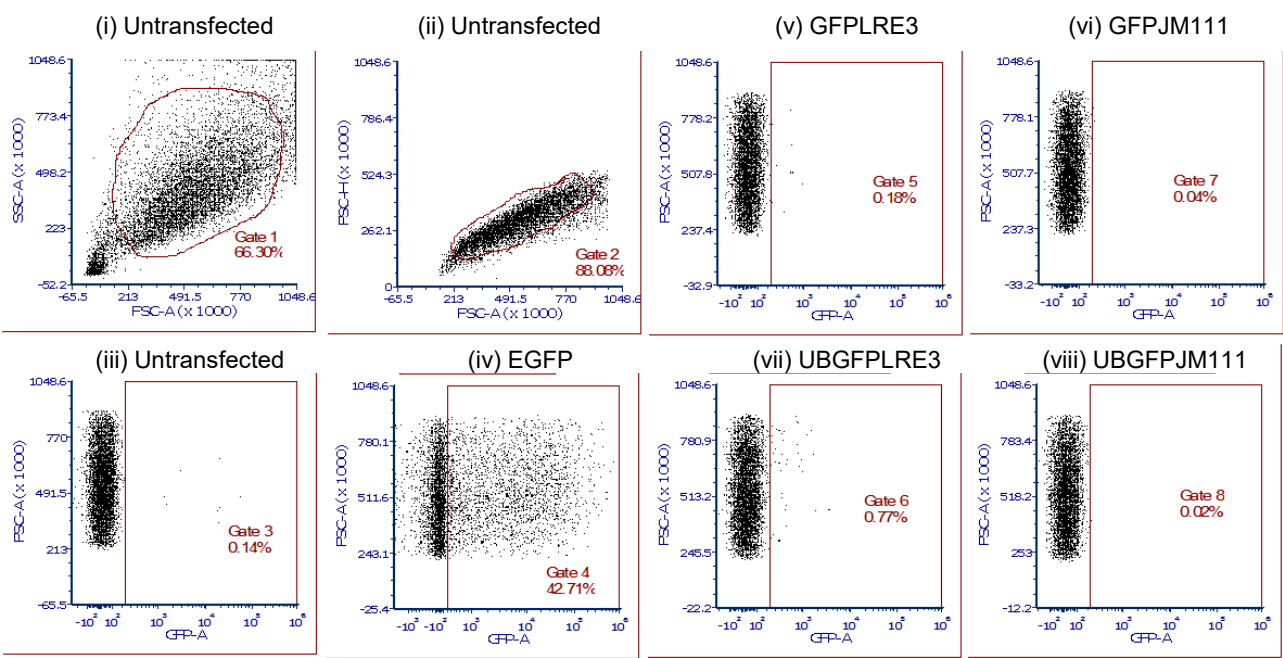

B

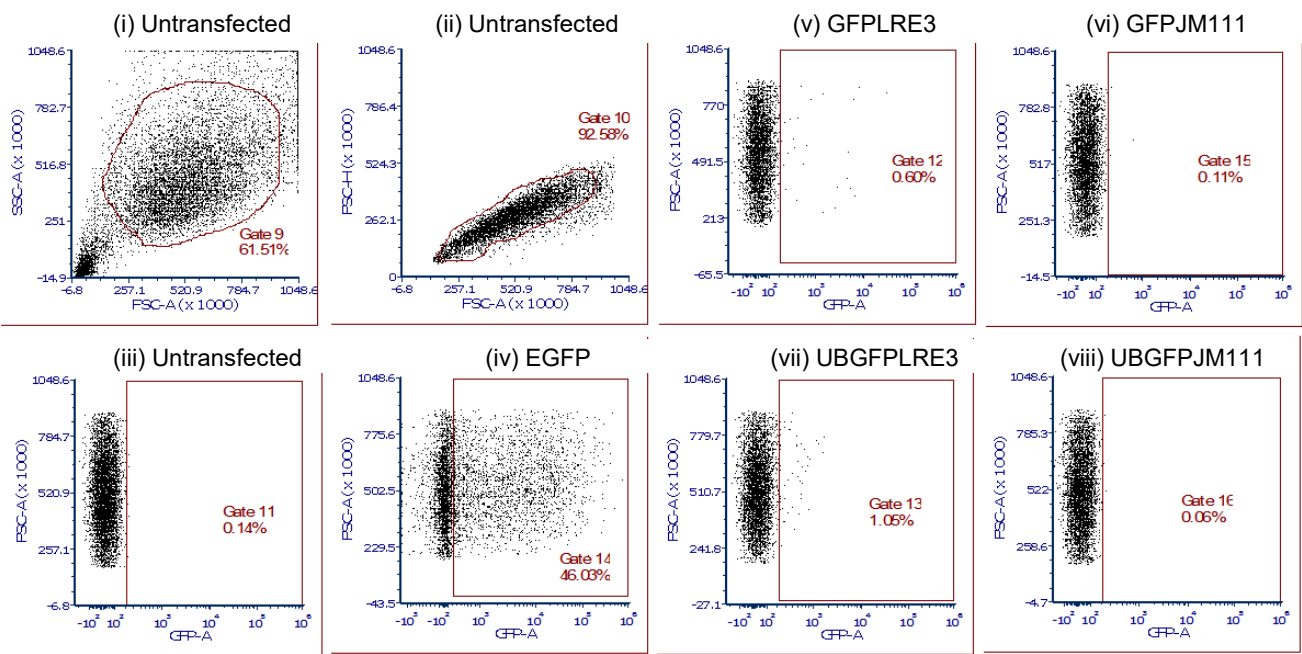

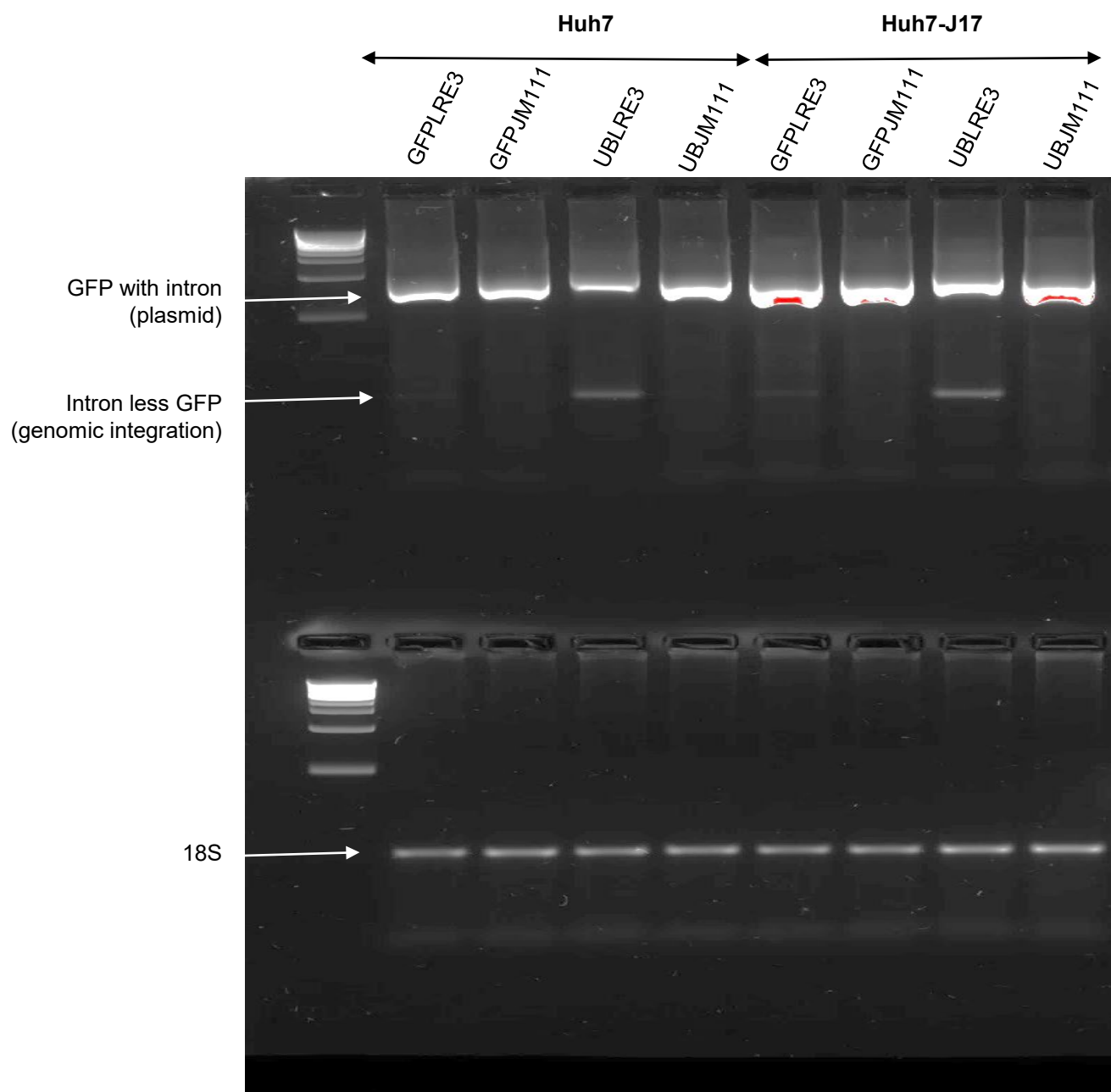

**A**

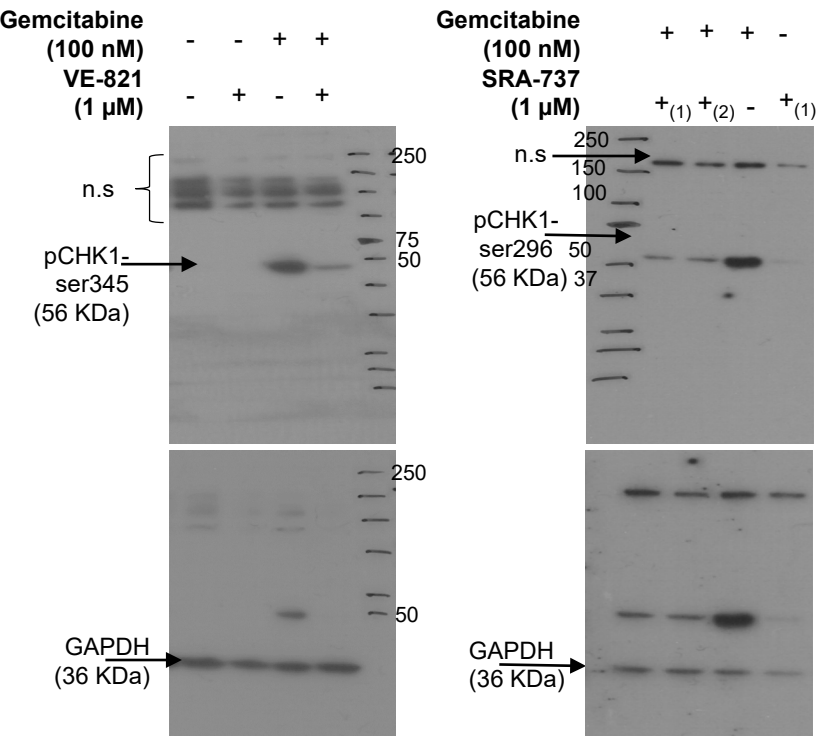

**B**

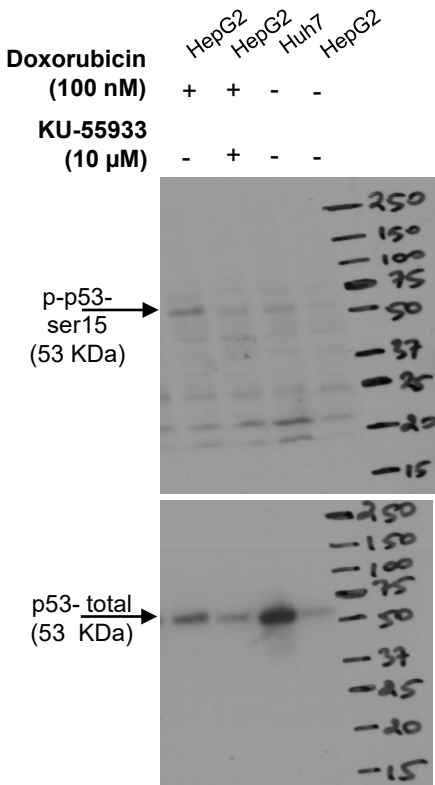

**C**

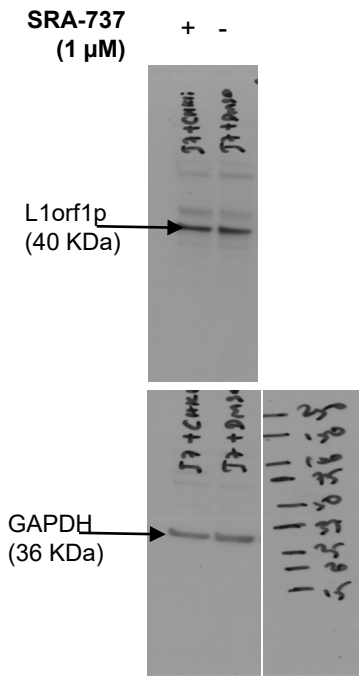

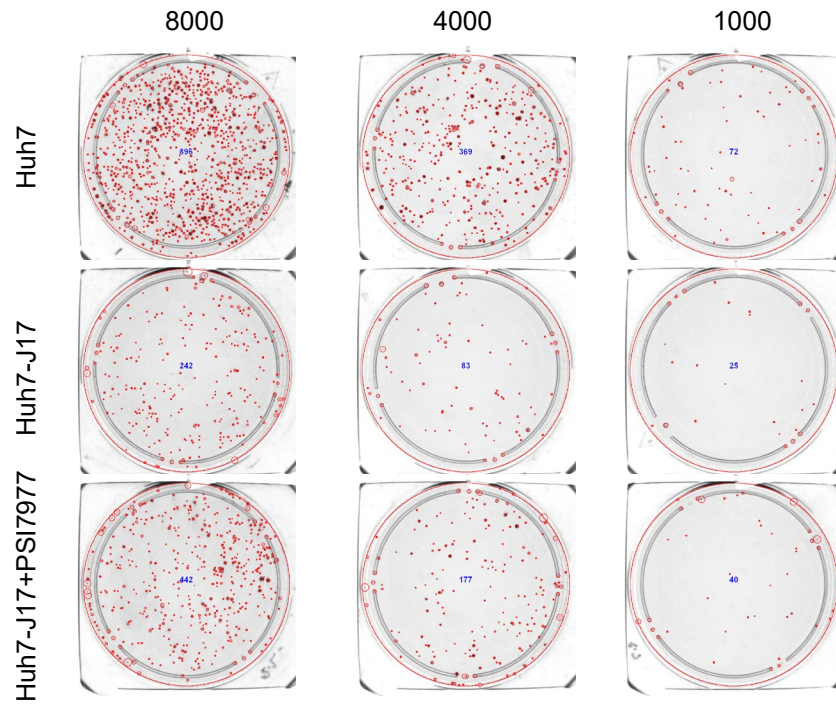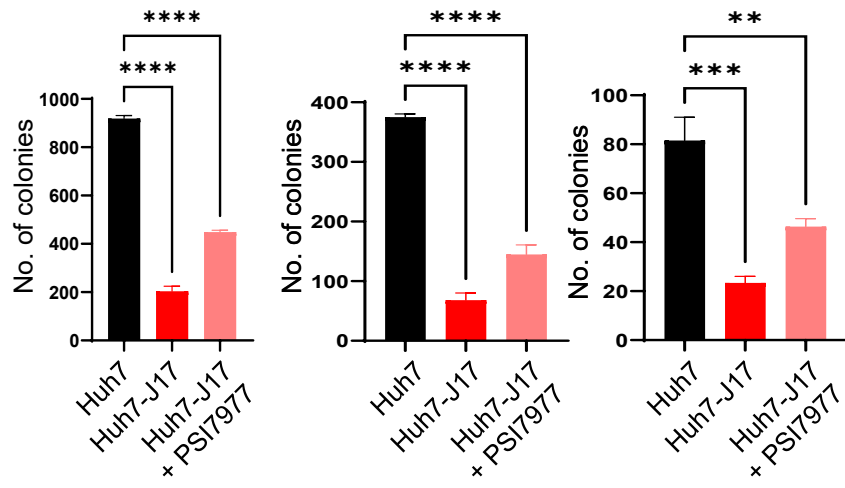

L1orf1p  
40 KDa

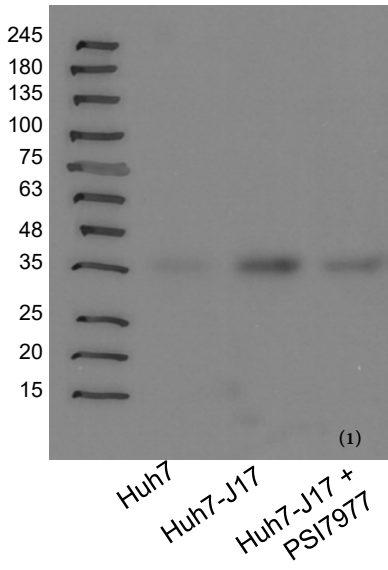

GAPDH  
36 KDa

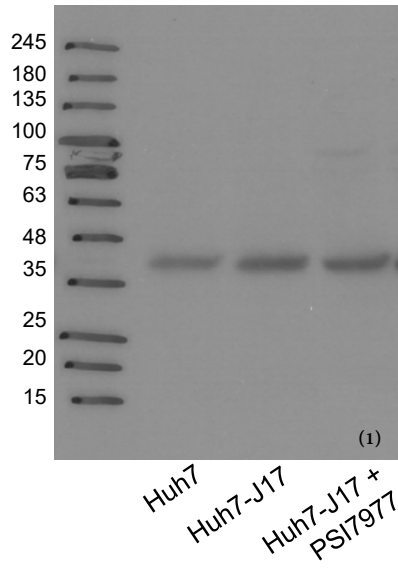

NS5A  
56 KDa

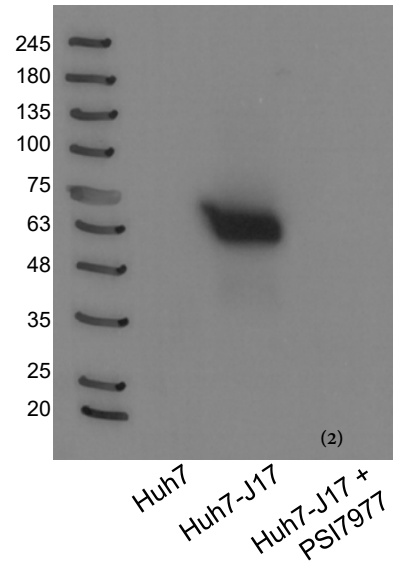

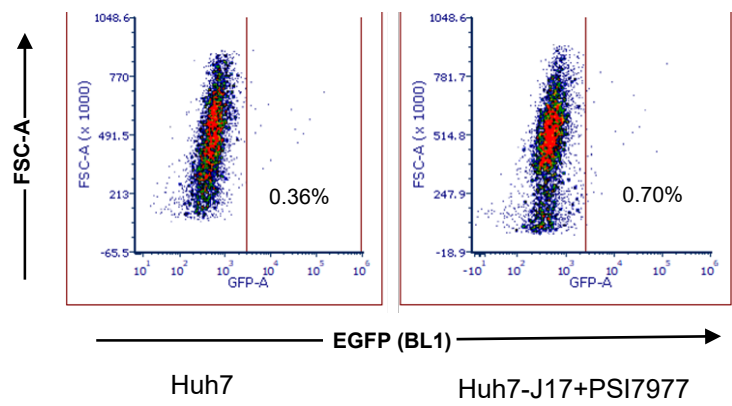

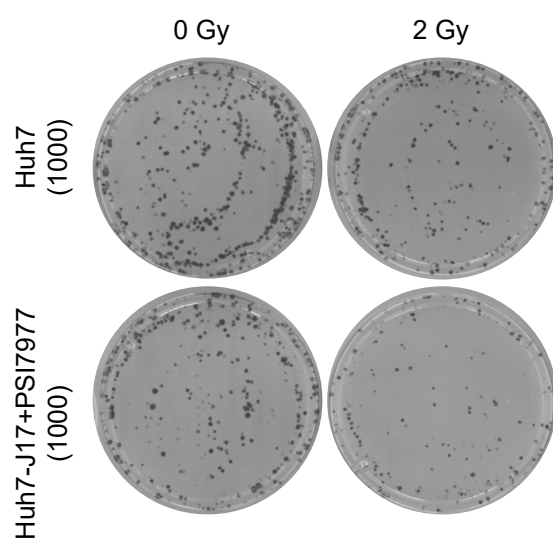

Supplement: Supplementary file 1 [file cancers-13-05079-s001.zip › cancers-1366777-figures-high resolution.pdf]
